# Supplementary material for: Genetic characterization of cucumber genetic resources in the NARO Genebank indicates their multiple dispersal trajectories to the East
Source: Theor Appl Genet. 2024 Jul 2;137(7):174. doi: 10.1007/s00122-024-04683-0 (PMC11219412; doi:10.1007/s00122-024-04683-0)
Supplement: Supplementary file 2 — Supplementary file2 (PDF 272 KB) [file 122_2024_4683_MOESM2_ESM.pdf]

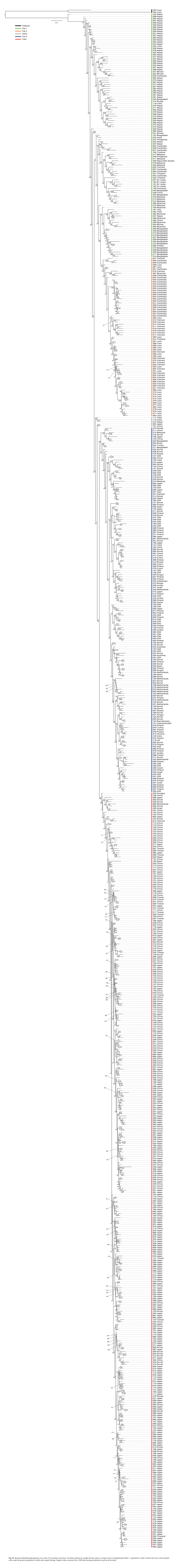

Fig. S1 Maximum-likelihood phylogenetic tree of the 723 cucumber accessions. Tip labels indicate the sample ID and country of origin shown in Supplemental Table 1, separated by a dash. Vertical line next to the tip labels color-codes the genetic population to which each sample belongs. Support values assessed from 1,000 ultra bootstrap replicates are given at the nodes

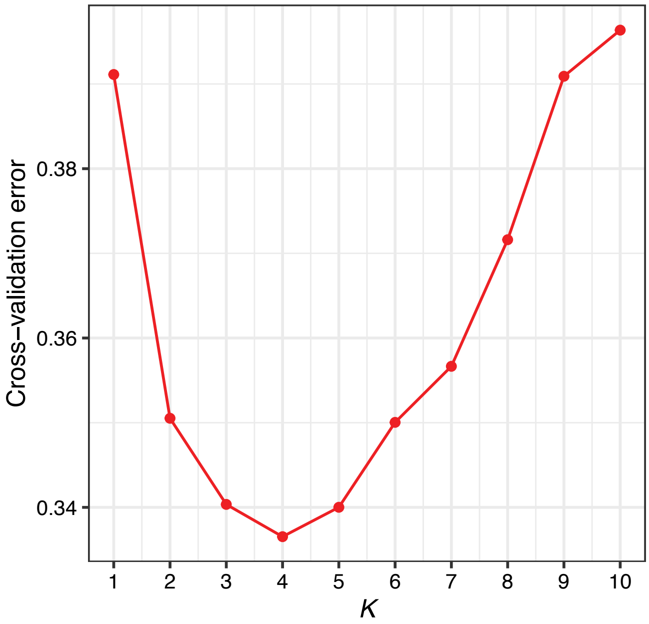

Fig. S2 Cross-validation errors for different values of assumed ancestral populations (*K*) from 1 to 10 in the ADMIXTURE analysis
